# Low-Cost Graphene-Based Digital Microfluidic System

**DOI:** 10.3390/mi11090880

**Published:** 2020-09-22

**Authors:** Mohamed Yafia, Amir M. Foudeh, Maryam Tabrizian, Homayoun Najjaran

**Affiliations:** 1 School of Engineering, University of British Columbia, Kelowna, BC V1V 1V7, Canada; 2 Department of Biomedical Engineering, Faculty of Medicine, McGill University, Montreal, QC H3A 0C7, Canada; amir.foudeh@mail.mcgill.ca (A.M.F.); maryam.tabrizian@mcgill.ca (M.T.); 3 Faculty of Dentistry, McGill University, Montreal, QC H3A 1G1, Canada

**Keywords:** digital microfluidics, graphene electrodes, laser scribing

## Abstract

In this work, the laser-scribing technique was used as a low-cost, rapid and facile method for fabricating digital microfluidic (DMF) systems. Laser-scribed graphene (LSG) electrodes are directly synthesized on flexible substrates to pattern the DMF electrode arrays. This facilitates the DMF electrodes’ fabrication process by eliminating many microfabrication steps. An electrowetting test was performed to investigate the effectiveness of the LSG DMF electrodes in changing the contact angles of droplets. Different DMF operations were successfully performed using the proposed LSG DMF chips in both open and closed DMF systems. The quality and output resolution were examined to assess the performance of such patterned electrodes in the DMF systems. To verify the efficacy of the LSG DMF chips, a one-step direct assay for the detection of *Legionella* *pneumophila* deoxyribonucleic acid (DNA) was performed on the chip without the need for any washing step. The high specificity in distinguishing a single-nucleotide mismatch was achieved by detecting target DNA concentrations as low as 1 nM. Our findings suggest that the proposed rapid and easy fabrication method for LSG DMF electrodes offers a great platform for low-cost and easily accessible point-of-care diagnostic devices.

## 1. Introduction

Microfluidic devices are recognized for being efficient platforms that can perform different chemical and biological assays [1,2,3,4]. Digital microfluidics (DMF) is one of the microfluidics platforms that is known for its ability to manipulate microliter droplets on discrete arrays of electrodes. One of the most important advantages of using this system is its ability to transport different droplets individually without using microvalves or mechanical moving parts [5].

Introducing rapid prototyping techniques has increased the number of published papers in the area of droplet-based microfluidics [6]. Consequently, several attempts have been initiated to fabricate the DMF platform at low cost to avoid using highly equipped facilities [7,8,9,10,11]. Photolithography is the conventional fabrication technique for DMF systems. Although this technique is able to generate features with high resolution, it requires a multiple-step fabrication process and well-trained individuals as well as sophisticated equipment and facilities. Conversely, rapid prototyping techniques provide the opportunity to fabricate DMF chips easily with modest equipment and in a more cost-effective manner. Some of these techniques enable printing the DMF-actuation electrodes on flexible substrates, modifying the electrode patterns and reprinting them, easily. This ability to reconfigure the electrode design is barely achievable with most conventional techniques. For example, screen printing is a rapid prototyping technique that is suitable for the low-cost and mass production of DMF chips, but it requires several steps to prepare and pattern electrodes [7].

In this work, a straightforward fabrication method is used where the electrodes are printed in a single step directly from computer aided design (CAD) software on a computer into the DMF chip using graphene. Graphene is one of the promising materials that has shown great potential for different applications [12]. It is characterized by having outstanding crystal properties, with a two-dimensional sheet structure that is only one atom thick [13]. Several biological applications are being developed with graphene-based platforms due to its biocompatibility [14,15]. However, graphene production is not a simple process [16]. Graphene commercialization has gained great interest globally, where many researchers are currently working on reducing its production cost [17,18,19]. Reducing graphene oxide (GO) is one of the techniques capable of producing reduced graphene oxide (rGO) layers [20]. Generally, photochemical (ultraviolet), photo-thermal (infrared) or laser reduction methods are used to reduce GO to graphene [21,22]. The conductivity of the GO layer before the laser-scribing process varies from 8.07 × 10^−4^ to 5.42 × 10^−3^ S/m. This conductivity increases significantly when GO is reduced to graphene, and it becomes 2.35 × 10^3^ S/m [23]. Currently, many applications that require graphene are taking advantage of the laser-scribing technique. To name a few, graphene-based electronic devices, such as in-plane transistors, photodetectors, loudspeakers and flexible strain sensors, have been fabricated using the laser-scribing technique [24,25]. Accordingly, the laser-scribing technique was used here to reduce graphene oxide to graphene for electrowetting-on-dielectric applications using readily available and simple equipment. 

The electrowetting phenomenon was discovered by G. Lippmann in 1875 [26]. Droplet manipulation and actuation were later demonstrated using electrowetting-on-dielectric (EWOD) by adding a dielectric layer and a hydrophobic layer on top of the electrodes, respectively [27]. Electrowetting-on-dielectric experiments were performed on graphene sheets fabricated by the chemical-vapor-depositing technique [28]. The graphene layers were then transferred to glass and polyethylene terephthalate (PET) substrates using advanced and sophisticated fabrication procedures. The authors performed an EWOD test and noticed that graphene exhibited higher capacitive impedance compared to gold. In addition, the use of graphene resulted in fewer defects and pinholes, reduced electrolysis and lowered the leakage current. The characterization of laser-scribed graphene electrodes was recently performed [29]. However, this work did not use the fabricated electrodes to demonstrate droplet motion on a digital microfluidic platform either in an open or in a closed DMF system, where adding a dielectric layer and hydrophobic layer is still necessary to demonstrate any successful DMF operation or biological application. 

**Figure 1:**
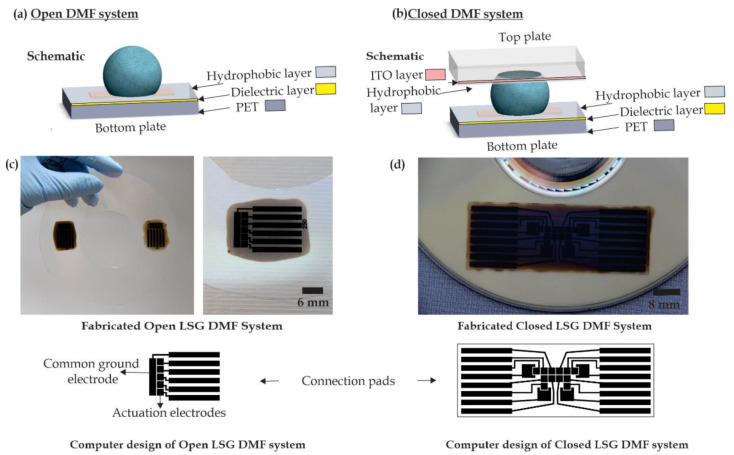
(**a**) Single-plate (open) digital microfluidic system versus (**b**) parallel-plate (closed) digital microfluidic system. (**c**) Two fabricated open-system digital microfluidic (DMF) chips printed with a long electrode acting as a common ground and five discrete electrodes for droplet manipulation. (**d**) A closed system with 12 electrodes and four reservoirs.

Digital microfluidic systems are characterized by having discrete electrodes, where EWOD is utilized to perform several operations on droplets at the microscale. Droplet transport, mixing, merging and dispensing have been demonstrated on the DMF platform [30,31]. In this work, we fabricated and tested DMF chips using laser-scribed graphene (LSG) electrodes in both open and closed system configurations. In open DMF systems, the droplet sits on a single plate where both the ground and the activated electrodes are located [32,33]. On the contrary, the droplet can be sandwiched between two plates in the closed DMF systems [34,35]. The difference in the construction of these two systems can be observed in Figure 1. Several dielectric layers and hydrophobic layers are added to the top and the bottom plates to functionalize the DMF chip and facilitate the droplet motion. 

Each of these systems has several advantages and disadvantages. Open systems are characterized by allowing easy access to the droplet and having higher droplet velocities and mixing rates. On the other hand, closed systems are characterized by having more controlled evaporation, as the droplets are sandwiched between two plates, where a filler medium can be used to minimize the evaporation. High-voltage electrical signals are usually used to transport the droplets on the electrode array. Open systems require higher voltages during operation (500–700 v) [36,37] compared to closed systems (25–200 v) [38].

In this work, we demonstrate the effectiveness of the fabricated DMF chips to help in detecting water contaminants. Water-borne disease outbreaks are a major concern in developed countries. *Legionella* is responsible for one third of these outbreaks. To date, there are more than 52 species of *Legionella* that have been identified; only half of these species are associated with human disease. In some cases, there is only one or two nucleotides of difference in their deoxyribonucleic acid (DNA) sequences. Accordingly, developing a highly specific detection method capable of distinguishing single-nucleotide mismatches is crucial. Here, we used a sophisticated molecular probe for this purpose, which consisted of a fluorescent dye and quencher at each end of molecular probe. In the absence of the target DNA, the fluorescent dye and quencher are in proximity to each other, which results in no fluorescent signal. When the target DNA hybridizes to this molecular probe, it becomes open and the fluorescent dye and quencher are situated at a distance from each other. By designing this molecular probe, there is no need to employ a washing step as with normal DNA-detector probes to wash away the excess of these probes. This is particularly advantageous for open DMF setups, in which the detection will happen in a single step by mixing the target DNA and molecular probe, and the results can be visualized with fluorescent microscopy.

In the present work, we used a simple method to fabricate graphene-based DMF systems. This fabrication method allowed us to generate graphene-based electrodes directly using a laser-scribing DVD burner on flexible and bendable PET substrates. Graphene is well known for its outstanding properties, and the laser-scribing technique is used for its low cost and versatility in creating different graphene patterns. For the proof of concept, the detection of single-nucleotide mismatches in *Legionella pneumophila* DNA was successfully demonstrated.

## 2. Materials and Methods

### 2.1. Laser Scribing Digital Microfluidics (DMF) Chips

A single-layer GO aqueous dispersion at a concentration of 5 mg/mL was purchased from Goographene (500 mL for 102 US Dollars) (Goographene, Merrifield, VA, USA). The sonication of the solution was performed for 1 h using an Ultrasonic Cleaner Branson 3510 (Fisher Scientific, Waltham, MA, USA) to ensure the complete dispersion of the graphene-oxide particles inside the solution. The 5 mg/mL GO solution was dispensed with a micropipette to ensure that the concentration of the dispensed GO volume per unit area was 0.57 µL/mm^2^. To cover the whole compact disc (CD) area, around 6 mL of GO are required, which approximately costs UD 1.2 per CD. This proves that the introduced fabrication method is cost effective with respect to the current graphene market prices, where these prices are expected to decrease significantly in the future [17,18,19]. The substrate was left to dry out for 24 h to form a uniform layer of GO. LightScribe CDs are different from normal CDs, as they have water marks near to the center for the accurate positioning of the CD during the laser-scribing process (Figure 1). 

**Figure 2:**
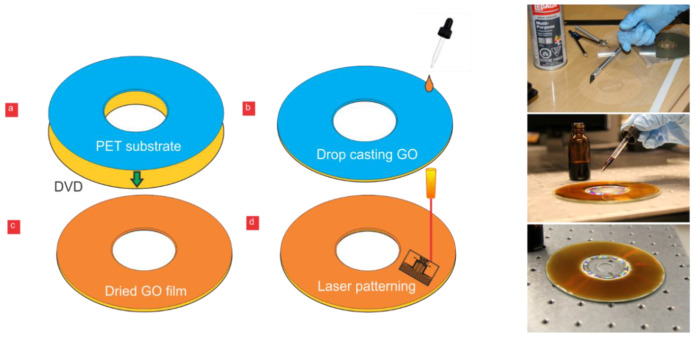
(**a**) polyethylene terephthalate (PET) substrate fixed on top of the LightScribe compact disc (CD). (**b**) Graphene oxide (GO) solution dispensed. (**c**) The substrate was left to dry for 24 h. (**d**) The CD was inserted into the laser-scribing drive, where the laser was used to form the required patterns. On the right-hand side are the steps for drop casting the graphene oxide on the PET substrate affixed on top of a CD. The PET substrate was cut and placed on a CD; then, the GO solution was drop casted and left to dry.

An LG GBC-H20L Blu-Ray Read DVD+RW Combo SuperMulti Drive with LightScribe was used in the laser-scribing process (LG, Seoul, South Korea). Memorex^TM^ LightScribe CDs were used in our experiments (Memorex 80 Minute 52X 10-Pack CD-R with LightScribe (Memorex, Cerritos, CA, USA)). We used a laser-scribing DVD drive to write on a laser-scribing CD. This laser-scribing device accepts both CDs and DVDs. The CD was covered with PET-3M transparency films (3M, Saint Paul, MN, USA). Two programs were tested for burning the required patterns on the CD: Nero Express 2014 (Nero AG, Karlsbad, Germany) and LightScribe Template Labeler (Hewlett-Packard, Palo Alto, CA, USA). Using the LightScribe Template Labeler software, we printed patterns on a bare laser-scribing CD, without attaching the PET with dried GO on top, with 50 µm spacings between the electrodes. The spacings were not visible, and the electrodes were completely connected to each other. The 50 µm electrode spacing became visible when we laser-scribed the same patterns using Nero Express. In addition, the overall contrast and sharpness were improved significantly. Unlike the LightScribe Template Labeler software, Nero Express provided more options for scaling the drawings and inserting the dimensions of the patterns accurately. An additional step that improved the darkness of the patterns was changing the contrast settings inside the LightScribe control panel from the default factory settings to darker labels with a longer labelling time. A PET sheet of 100 µm thickness (3M) was used as a substrate and was fixed on top of the CD surface. The graphene-oxide solution was added on top of the PET surface by drop casting. The PET substrate was fixed on the top of the CD surface with an adhesive spray (LePage Pres-Tite Multi-Purpose Spray Adhesive). The CD that held the dried graphene oxide on top of the PET substrate was inserted into the LightScribe DVD drive, where the patterns were laser scribed to generate conductive graphene. The laser followed the designed patterns to reduce the graphene-oxide layer to conductive graphene. Figure 2 demonstrates the sequence of the previous steps. The LSG graphene electrodes were covered with a 10 µm layer of Parylene-C by chemical-vapor deposition to form the dielectric layer (Specialty Coating Systems, Indianapolis, IN, USA). Teflon AF1600 (Dupont, Wilmington, DE, USA) was spin-coated on top to act as a hydrophobic layer. We used the following spin coater: Laurell WS-650-23 (Laurell Technologies Corporation, North Wales, PA, USA). The spin speed used was 2000 RPM for 30 s. The voltage was applied with a Trek Model PZD700A high-voltage power amplifier/piezoelectric driver (Trek, Lockport, NY, USA). The contact angle was measured using an Image J plugin called DropSnake. The resistance was measured with an Amprobe 38XRA (Amprobe, Everett, WA, USA). The droplet velocity was measured with a high-speed camera (HiSpec 5 High-Speed Camera, Fastec Imaging, San Diego, CA, USA) and an open-source motion-analysis software (Tracker Video Analysis and Modeling Tool).

### 2.2. Molecular-Probe Design and Hybridization Assays

Oligonucleotides were purchased from Integrated DNA Technologies (Coralville, IA, USA). DNA capture probes (CP), complementary to *L. pneumophila*‘s 16s rRNA, were selected and designed using Proimose [39] and OligoArchitect Online from Sigma-Aldrich. For hybridization, a 10 mM Tris-HC (pH 8.0) solution with 1mM MgCl_2_ was used. A fluorescence microscope (Leica Z16 APO, Concord, ON, Canada) coupled with a fluorescence illuminator (X-Cite^®^ series 120 Q, Excelitas Technologies Corp., Waltham, MA, USA) was used for the measurement of the fluorescence intensity of the droplets on the chip. All the images were captured using a charged-coupled device (CCD) camera (Leica DFC340 FX, Concord, ON, Canada) and analyzed with ImageJ (National Institutes of Health, Bethesda, MD, USA). The intensity obtained from a negative control was subtracted from all the measurements. The negative-control droplet contained molecular beacons. The lower detection limit was defined as the smallest concentration of an analyte, calculated as the blank signal plus or minus three standard deviations. All the data are expressed as the mean ± standard deviation.

## 3. Results

### 3.1. Electrowetting, Droplet-Actuation and Printing-Resolution Tests

**Figure 3:**
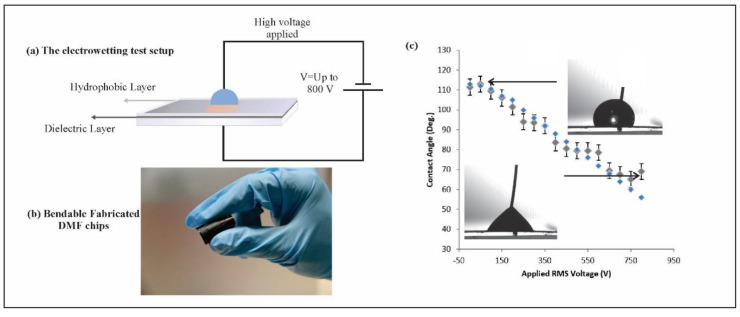
(**a**) The electrowetting test setup. (**b**) The fabricated chips were bendable, which could allow the droplets to move in inclined, twisted and vertical directions. (**c**) The electrowetting test performed to characterize the change in the contact angle. Blue dots represent the trend line, where the contact angle saturation occurred at 70 degrees.

An electrowetting test was performed to determine if this new system was capable of changing the droplet contact angle and to characterize the change in the contact angle at different voltages. Figure 3 depicts that the fabricated chip was able to manipulate the contact angle successfully when the applied voltage was increased to 800 v.

The DMF platform fabricated using the laser-scribing technique is shown in Figure 1. In this figure, the LSG electrodes are demonstrated for open and closed systems. In closed systems, on-chip reservoirs are arranged around the electrode array for performing closed DMF operations. A top plate of indium tin oxide (ITO) covered with a hydrophobic layer was added to act as a grounding system. In all the previous studies, the GO solutions were drop casted to completely cover the whole circular area on the CD in order to print small structures repeatedly, such as transistors [24], all over the CD printable area and prevent wasting a large area of unused GO. However, the DMF chips occupied relatively larger areas compared to the previously reported structures in [24] and could not be repeated easily beside each other. This would generate large wasted areas of GO on the CD surface area. Accordingly, we dispensed the GO solution only on the designed DMF chip area to prevent wasting large amounts of unused GO area. The amount of the dried GO on the surface depends on the dispensed volume of the GO solution and its concentration. In this work, the amount of GO per unit area used was 0.57 mg/mm^2^. We can observe in Figure 1 that the GO solution was only added to the rectangular section of the chip instead of covering the whole CD surface. Using PET as a flexible substrate can enhance the capabilities of the LSG DMF chip. Figure 3b shows that the fabricated DMF chips were flexible and easily bendable. This could allow the droplets to move in inclined, twisted and vertical directions [36]. 

**Figure 4:**
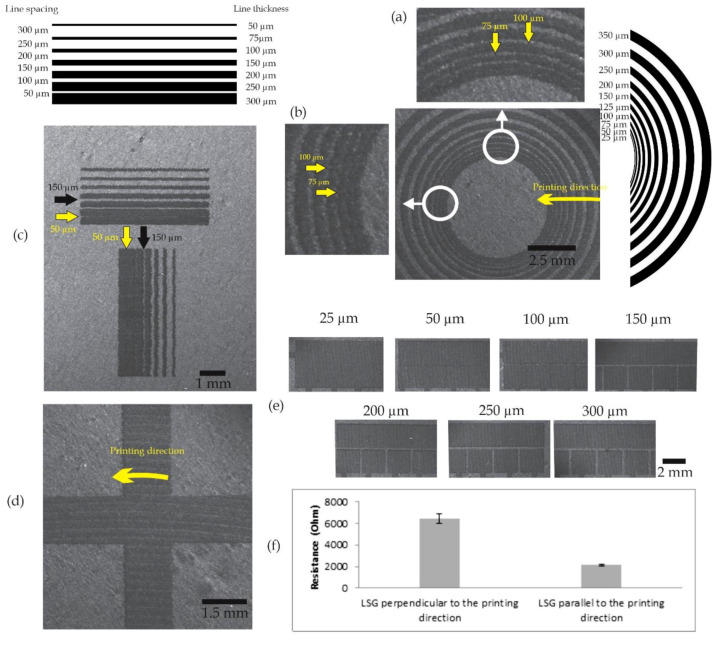
Printing concentric circles with resolutions increasing from 25 to 350 µm to assess and characterize the printing quality in all the directions and angles in (**a**,**b**). (**c**) Printing resolution test on vertical and horizontal lines. (**d**) Two long electrodes printed in the vertical and horizontal directions, 15 × 1.6 mm, to investigate the effect of the printing direction on the measured resistance in (**f**). (**e**) DMF electrodes printed at different resolutions. (**f**) The resistance of the two laser-scribed graphene electrodes in the horizontal direction (parallel to the printing direction) and the vertical direction (perpendicular to the printing direction).

It was important to determine the minimum attainable printing resolutions and investigate the limitations and the capabilities of the laser-scribing technique. Several tests were performed to characterize the output resolution of the LSG DMF electrodes since the printing resolution is critical for fabricating the functional electrodes required for DMF applications. During the laser-scribing process, the laser passed in circular patterns on the dried GO. Consequently, the graphene layers were not formed uniformly, and concentric circular patterns could be seen clearly on the surface (see Figure 4) due to the printing path. In order to characterize the uniformity of the printed patterns, two tests were performed to discover how printing in a circular manner could affect the spacings between the electrodes and the line thickness of the connection lines.

In the first test, concentric circles were printed with equal line thickness and spacings, from 25 to 350 µm. Noticeable differences could be observed in the thickness of the printed lines and the spacings between them in different directions. Figure 4a,b show that the lines were completely connected for a nominal designed spacing of 25 µm at the first circle, and this spacing disappeared completely in the actual printed pattern. In Figure 4a, the line spacing at 50 and 75 µm started to become visible and that at 100 µm became well defined. However, in Figure 4b, it can be observed that the 25, 50 and 75 µm lines were connected and the 100 µm line was visible; starting from 125 µm, the lines became more defined. Thus, we verified that the printing process is not uniform in all directions and the printing resolution is affected by the position of the patterns placed and the printing direction.

In the second test, parallel graphene lines were printed in the horizontal and the vertical directions to assess the printing resolution and the electrode spacings in these two main directions. Figure 4c shows that the nominal line spacings were increased from 50 to 300 µm. Conversely, the line thickness was decreased from 300 down to 50 µm in the opposite direction. The horizontal lines were tangential to the circular-printing direction, and the vertical lines were perpendicular to the circular-printing direction, which was the same as the printing direction shown in Figure 4d. When the nominal designed spacing became 50 µm between the first two vertical lines from the left, we can observe that the actual printed lines became completely connected and the spacing disappeared. On the other hand, the first two horizontal lines from the top were not completely connected, where very small gaps started to appear at the same theoretical 50 µm spacing. At 150 µm spacing, the lines in the horizontal direction were more defined and well separated compared to the lines in the vertical direction, which were still connected at some points. We can observe that the printed lines were smooth and more defined in the horizontal lines compared to the corrugated and more-connected vertical lines in Figure 4c.

To verify the previous results for the square-shaped DMF electrodes, a printing test was performed on an actual arrangement of fabricated LSG DMF electrodes. In this printing test, the line spacings between the electrodes were increased from 25 to 300 µm (Figure 4e). Similarly, we can observe that the electrodes were completely connected at 25 µm, and they started to become more separated when the resolution increased, following the same previous pattern with the printed lines.

Accordingly, we concluded from these experiments that we have to use 250 µm spacing in the next experiments to mitigate all the previous irregularities in the line spacings. This relatively large spacing will not hinder the DMF operations, as we were able to perform DMF operations when the line spacing between the electrodes went up to 300 µm [7]. To investigate if the printing direction could affect the resistance on relatively large electrodes, we measured the resistance of the vertical and horizontal printed lines presented in Figure 4d. Figure 4f depicts that the electrical resistance of the printed line in the vertical direction was relatively higher than the resistance in the horizontal direction. This difference can be explained if we look closely at the printing patterns engraved on the surface of the two printed lines in Figure 4d. In case of the vertical line, a large number of small curves and small discontinuities were formed perpendicular to the vertical printed line. The transition to this large number of curves resulted in larger resistance. On the other hand, the horizontal lines encountered fewer transition curves tangential to the printing direction, which resulted in lower overall resistance. 

### 3.2. Performing DMF Operations on the Laser-Scribed Graphene (LSG) DMF System

**Figure 5:**
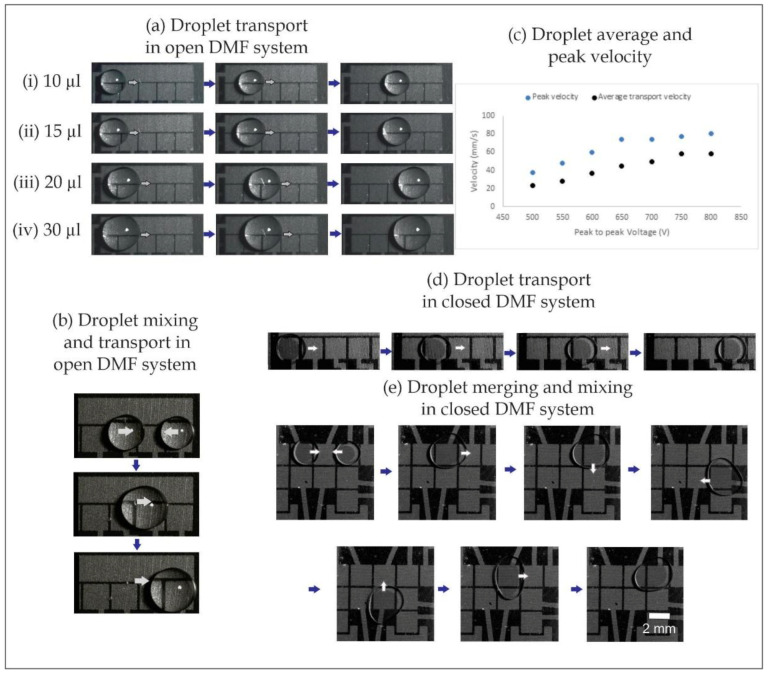
(**a**) Droplet-transport experiments with various droplet volumes in open DMF systems (without a top cover). (**b**) Two droplets were merged and mixed; then, the bigger droplet was transported. (**c**) The peak and averaged droplet velocities measured from the experiments. (**d**,**e**) Transport, merging and mixing of droplets on laser-scribed graphene-based DMF systems in a closed DMF configuration (with top cover acting as a ground electrode).

Open and closed LSG DMF systems were successfully fabricated based on the previous characterization results to ensure functional DMF electrodes. The fabricated open and closed LSG DMF systems were able to manipulate various droplet volumes successfully, as shown in Figure 5. The fabricated open DMF chip, with a 2 mm electrode size and 250 µm electrode spacing, was able to manipulate a wide range of droplet volumes, from 10 µL up to 30 µL. Smaller droplets can be transported in an open system configuration using a smaller electrode size if needed. As shown in Figure 5b, we merged and mixed two droplets and then transported the resultant bigger droplet to show that the same electrode size could handle various ranges of droplet volumes. To characterize the chip performance, the average droplet velocity and the peak droplet velocity also were plotted against the voltage used (AC square wave signal with a frequency of 10 kHz). The droplet velocity ranged from 20 to 80 mm/s, which falls within the values previously reported in the literature [40,41]. However, the velocities in the present work cannot be directly compared to those in other studies, as we used a different electrode size, electrode shape, grounding configuration, dielectric material, dielectric thickness and voltage, which can generate different actuation forces [37]. The droplet displacement and velocity are plotted against time at 500 V in the Figures S1 and S2. The droplet-velocity profile was calculated from the droplet-displacement data measured by a high-speed camera at 300 frames per second. The average droplet velocity was calculated by dividing the total droplet displacement by the total displacement time. The droplet peak velocity was measured during the bulk motion of the droplet and not during the initial acceleration stage when the electrowetting forces were only acting on the droplet meniscus [42].

To test the LSG DMF electrodes in a closed DMF system configuration, a transparent ITO ground plate coated with a hydrophobic layer was added on top. As shown in Figure 5d, a 4 µL droplet was transported successfully using 250 V at a frequency of 10 kHz. Furthermore, another test was performed to demonstrate the process of merging two droplets, 2 µL each. After merging the droplets, they were mixed and then transported as one 4 µL droplet over the electrode array in a square-shape mixing pattern, as shown in the sequenced images in Figure 5e.

### 3.3. Validation of the DMF Device Using a Single-Nucleotide Mismatch Discrimination Assay

**Figure 6:**
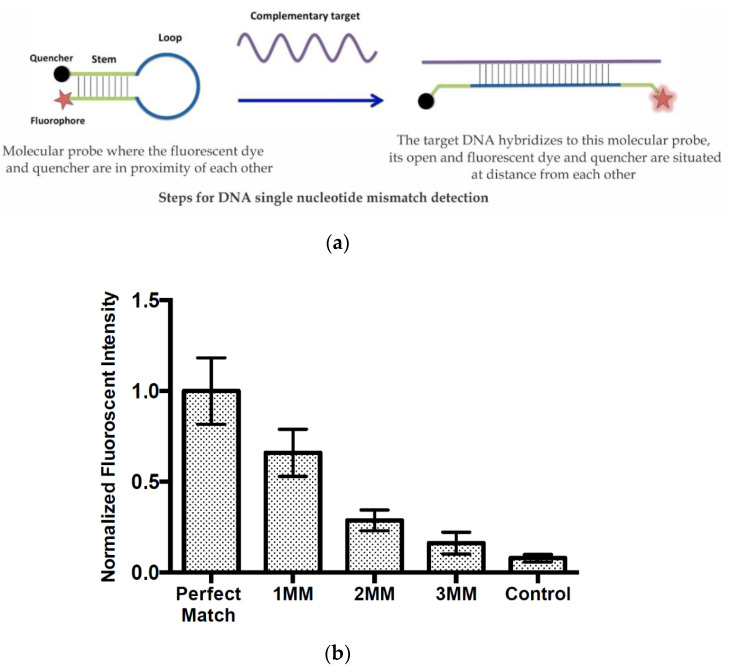
(**a**) The steps of the stretching process for the designed molecular probe during the process of DNA mismatch (MM) detection. (**b**) Normalized fluorescent intensity corresponding to perfect match, 1–3 mismatches and control.

To demonstrate the applicability of the LSG in point-of-care applications, we further demonstrated a one-step direct assay for the detection of *L. pneumophila* DNA without the need for any washing step, with high specificity for discriminating a single-nucleotide mismatch in the DNA sequences shown in Table 1. This was achieved by designing a molecular beacon with a fluorophore and quencher in proximity in the closed state. In Figure 6b, the normalized fluorescent intensity was plotted for 1 to 3 mismatches in addition to for a perfect match and control sample. This clearly demonstrated that the designed assay was highly sensitive to the target sequence and was capable of distinguishing a single mismatch. In order to emphasize the signal difference between the perfect match and other mismatches, the fluorescence intensity was normalized based on the perfect mismatch sample. The droplets were merged in an open DMF system, as the mixing occurred once the droplets were merged together. Open DMF systems are known to be better than closed systems in mixing, as the droplet needs to be moved around electrodes several times for mixing to occur in closed DMF systems. Droplets of 10 µL were mixed and incubated for 5 min. The portable, low-cost and easy-to-manufacture DMF chips in combination with one-step and highly specific molecular probes offers a great platform for point-of-care diagnostics. In order to obtain high specificity, in addition to the considerations in designing molecular beacons, the hybridization conditions in particular salt concentrations are critical [43]. Here, we examined several salt concentrations since the cations affect the stability of hybridized oligonucleotides, and a 10 mM Tris-HC solution with 1 mM MgCl_2_ resulted in the highest specificity. In order to demonstrate the selectivity of the designed molecular beacon targeting *L. pneumophila’s* DNA, several DNA targets complementary to the molecular beacon with 1 to 3 mismatches were designed. As can be seen in Figure 6b, there was a clear signal reduction upon increasing the number of mismatches. This is due to the fact that by introducing mismatches, the stability of the molecular beacon–target duplex was reduced (due to the lowering of the duplex melting temperature), and this resulted in fewer molecular beacons in the opened state (the quencher remained in proximity to the fluorophore) and therefore less fluorescent signal.

**Figure 7:**
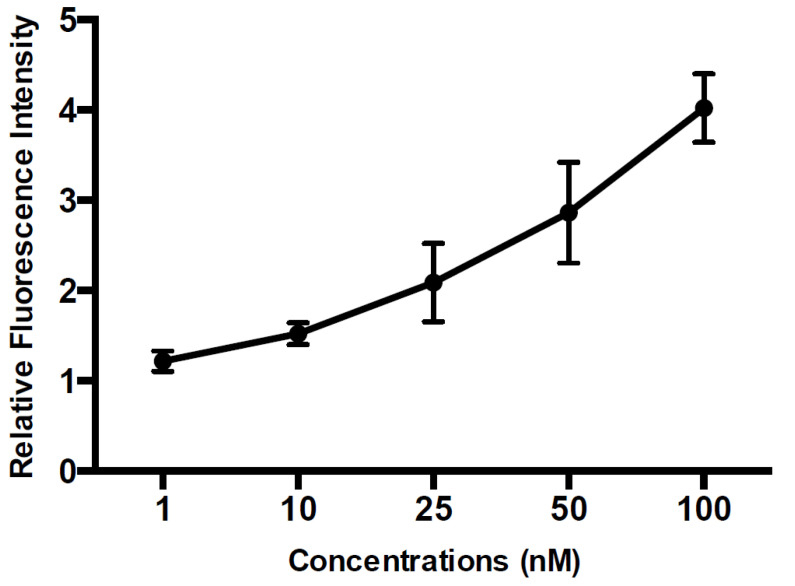
Measured relative fluorescence intensity according to *Legionella* deoxyribonucleic acid (DNA) concentrations. The relative fluorescence intensity is the ratio of the sample fluorescent intensity to that of the control.

The probe concentration used was 100 nM. Our results suggested that, with the design of the molecular beacon and careful selection of the hybridization buffer, we were able to demonstrate a highly specific probe that could distinguish single-nucleotide mismatches. In order to determine the working range of our detection system, several concentrations of the target DNA were hybridized with the molecular beacon. As shown in Figure 7, we were able to detect target DNA concentrations as low as 1 nM.

## 4. Discussion and Conclusions

In this work, low-cost graphene electrodes were used to perform various digital microfluidic operations in open and closed DMF systems. The fabrication method involved the laser-scribing of graphene to generate DMF electrodes on flexible substrates directly from a CAD design without the need for cleanrooms and microfabrication facilities. Several tests were conducted to characterize the LSG-fabricated DMF chips and to evaluate the performance of the LSG-based chips. The printing resolution of the LSG DMF chips was characterized, and the horizontal and vertical printed graphene lines appeared to be slightly different due to the circular laser-scribing paths of the device used. The printing resolution has to be more than 250 µm to make sure that there is a reasonable spacing between the electrodes and that they are not connected across the horizontal and vertical directions. The LSG DMF electrodes substantially changed the contact angle of the droplet via EWOD. Several DMF operations were introduced in both the closed and the open DMF to transport, merge and mix small droplet volumes, from 2 µL up to 30 µL. This platform had the capability of detecting *L. pneumophila* DNA with a limit of detection of 1 nM and with high specificity at the level of a single-nucleotide mismatch. Our findings suggest that the developed DMF device can be used in portable, low-cost and versatile platforms for the one-step detection of molecular probes.

## Figures and Tables

**Table 1:** DNA sequences used in the experiments.

Names	Sequences
Molecular Probe	Fluorescein*/CGAGCC* ATTATCTGACCGTCCCA *GGCTCG*/Iowa Black FQ
Perfect match	TGGGACGGTCAGATAAT
1 MM	TGGGACGG**A**CAGATAAT
2 MM	TGGGACG**AA**CAGATAAT
3 MM	TGGGACG**AAT**AGATAAT

MM: Mismatch.

## Supplementary Materials

The following are available online at https://www.mdpi.com/2072-666X/11/9/880/s1. Figure S1: The droplet displacement plotted against time when 500 V is applied, Figure S2: The droplet instantaneous velocity plotted against time when 500 V is applied.
