# Supplementary material for: Low-Cost Graphene-Based Digital Microfluidic System
Source: Micromachines (Basel). 2020 Sep 22;11(9):880. doi: 10.3390/mi11090880 (PMC7569958; doi:10.3390/mi11090880)
Supplement: Supplementary file 1 [file micromachines-11-00880-s001.pdf]

Supplementary Material

Low-Cost Graphene-Based Digital Microfluidic System

Mohamed Yafia, Amir M. Foudeh, Maryam Tabrizian and Homayoun Najjaran

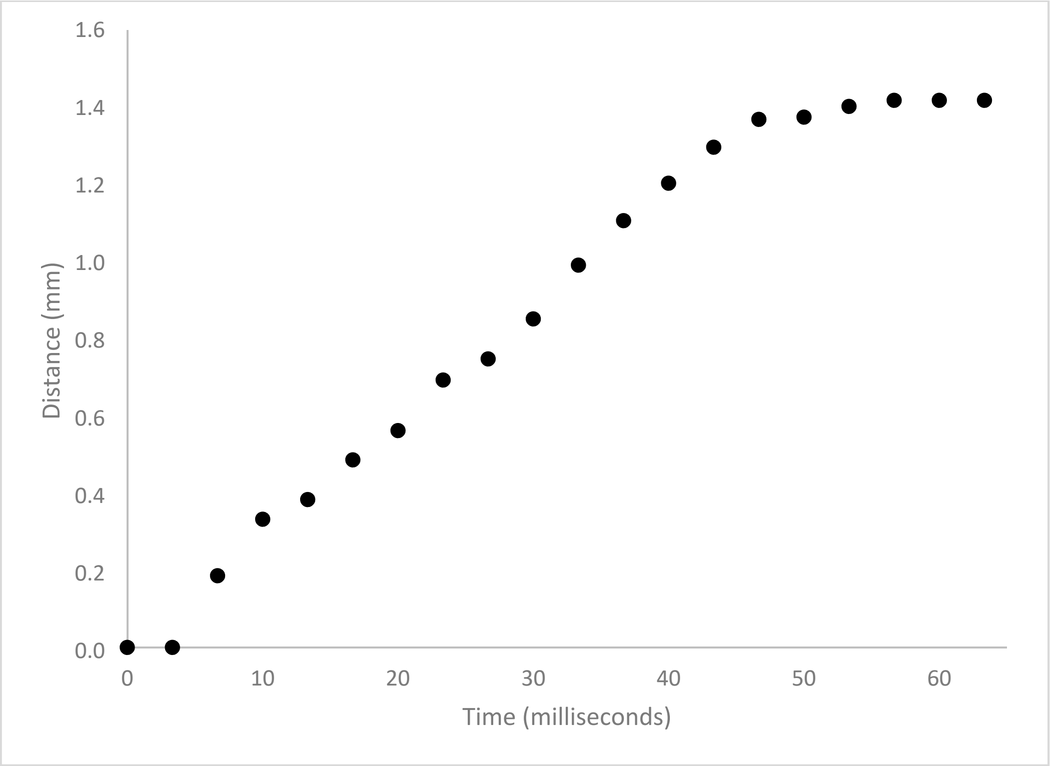

Figure S1. The droplet displacement plotted against time when 500 V is applied.

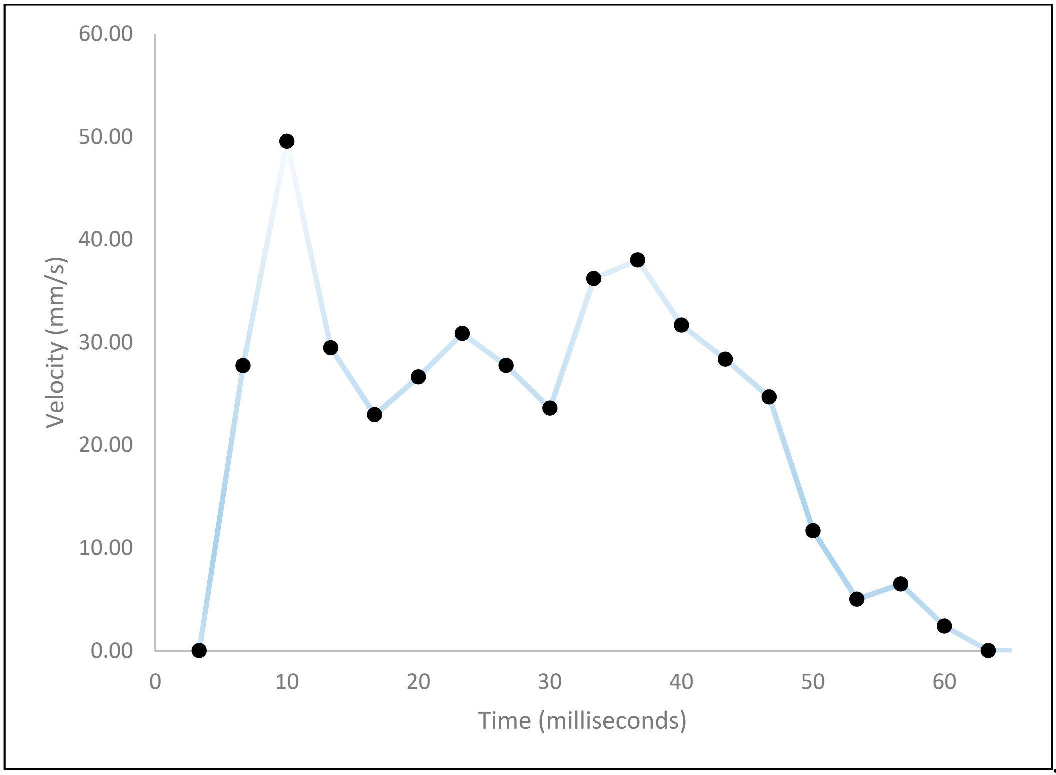

Figure S2. The droplet instantaneous velocity plotted against time when 500 V is applied.

The average velocity is calculated by dividing the total displacement distance by the total displacement time in Figure S1. The droplet peak velocity is the maximum droplet velocity during the droplet bulk motion which happens at the second peak in Figure S2. The first peak is representing only the velocity of droplet meniscus during the initial stages of the motion where the whole droplet is still stagnant and the droplet meniscus is stretched toward the activated electrode.

© 2020 by the authors. Submitted for possible open access publication under the terms and conditions of the Creative Commons Attribution (CC BY) license (http://creativecommons.org/licenses/by/4.0/).
